# Population pharmacokinetics of primaquine and its metabolites in African males

**DOI:** 10.1186/s12936-024-04979-y

**Published:** 2024-05-21

**Authors:** Palang Chotsiri, Almahamoudou Mahamar, Halimatou Diawara, Pius S. Fasinu, Kalifa Diarra, Koualy Sanogo, Teun Bousema, Larry A. Walker, Joelle M. Brown, Alassane Dicko, Roly Gosling, Ingrid Chen, Joel Tarning

**Affiliations:** 1 Mahidol Oxford Tropical Medicine Research Unit, Faculty of Tropical Medicine, Mahidol University, 420/6 Rajvithi Road, Bangkok, 10400 Thailand; 2 Malaria Research and Training Centre, Faculty of Pharmacy and Faculty of Medicine and Dentistry, University of Sciences Techniques and Technologies of Bamako, Bamako, Mali; 3 Department of Pharmacology & Toxicology, University of Alabama at Birmingham, Birmingham, AL 35294 USA; 4 Department of Medical Microbiology, Radboud University Medical Center, Nijmegen, The Netherlands; 5 National Center for Natural Products Research, The University of Mississippi, University, MS USA; 6 Department of Epidemiology and Biostatistics, University of California, San Francisco, CA USA; 7 Department of Disease Control, London School of Hygiene and Tropical Medicine, London, UK; 8 Malaria Elimination Initiative, University of California, San Francisco, CA USA; 9 Centre for Tropical Medicine and Global Health, Nuffield Department of Medicine, Oxford University, Oxford, UK

**Keywords:** Primaquine, Carboxy-primaquine, Primaquine carbamoyl-glucuronide, Pharmacokinetics, Nonlinear mixed-effect model, G6PD-deficiency

## Abstract

**Background:**

Primaquine (PQ) is the prototype 8-aminoquinoline drug, a class which targets gametocytes and hypnozoites. The World Health Organization (WHO) recommends adding a single low dose of primaquine to the standard artemisinin-based combination therapy (ACT) in order to block malaria transmission in regions with low malaria transmission. However, the haemolytic toxicity is a major adverse outcome of primaquine in glucose-6-phosphate dehydrogenase (G6PD)-deficient subjects. This study aimed to characterize the pharmacokinetic properties of primaquine and its major metabolites in G6PD-deficient subjects.

**Methods:**

A single low-dose of primaquine (0.4–0.5 mg/kg) was administered in twenty-eight African males. Venous and capillary plasma were sampled up to 24 h after the drug administration. Haemoglobin levels were observed up to 28 days after drug administration. Only PQ, carboxy-primaquine (CPQ), and primaquine carbamoyl-glucuronide (PQCG) were present in plasma samples and measured using liquid chromatography mass spectrometry. Drug and metabolites’ pharmacokinetic properties were investigated using nonlinear mixed-effects modelling.

**Results:**

Population pharmacokinetic properties of PQ, CPQ, and PQCG can be described by one-compartment disposition kinetics with a transit-absorption model. Body weight was implemented as an allometric function on the clearance and volume parameters for all compounds. None of the covariates significantly affected the pharmacokinetic parameters. No significant correlations were detected between the exposures of the measured compounds and the change in haemoglobin or methaemoglobin levels. There was no significant haemoglobin drop in the G6PD-deficient patients after administration of a single low dose of PQ.

**Conclusions:**

A single low-dose of PQ was haematologically safe in this population of G6PD-normal and G6PD-deficient African males without malaria.

*Trial registration* NCT02535767

**Supplementary Information:**

The online version contains supplementary material available at 10.1186/s12936-024-04979-y.

## Background

Primaquine (PQ) is a widely available anti-malarial that kills the dormant liver stage (hypnozoites) of *Plasmodium vivax* and *Plasmodium ovale* [1]. The World Health Organization (WHO) suggests a daily treatment (i.e., 0.25 mg/kg daily for 14 days) for glucose-6-phosphate dehydrogenase (G6PD)-normal individuals and a weekly treatment (i.e., 0.75 mg/kg weekly for 8 weeks) for G6PD-deficient individuals. PQ also possesses a sterile effect against the sexual stage of the *Plasmodium* parasite. A single low dose (SLD) of PQ with a standard artemisinin-based combination therapy (ACT) has shown gametocyte reduction and transmission blocking properties [2–4]. Therefore, the WHO recommends a 0.25 mg/kg SLD-PQ in all *Plasmodium falciparum*-infected patients living in the area approaching malaria elimination and/or facing drug resistance [1, 5].

Pharmacokinetic properties of PQ have been well characterized. PQ is almost completely absorbed (96% bioavailability) and rapidly eliminated (5–6 h terminal half-life). PQ can be metabolized via several pathways including the activities of monoamine oxidase (MAO-A), cytochrome P450 (CYP) isoenzyme, and uridine 5ʹ-diphospho-glucuronosyltransferase (UDP-glucuronosyltransferase, UGT). Also, its metabolism and pharmacokinetic profiles is enantioselective [6, 7]. Carboxy-primaquine (CPQ) is the major metabolite found in human plasma and is generated via the MAO-A-mediated pathway. The pharmacological effects of PQ have been attributed to its metabolites, and although several metabolites of PQ can be detected in human plasma and urine, those responsible for the biologic activity are still poorly understood [8, 9]. Presumably, the quinone-imine and orthoquinone metabolites were proposed as the active metabolites, because they can generate local reactive oxygen species (ROS) through a redox reaction which can results in oxidative damage to the parasites and potentially cause haemolysis of the red blood cell [10, 11].

A major adverse effect of 8-aminoquinoline drug is the drug-induced haemolytic effect especially in G6PD-deficient individuals. Different variants of the G6PD genotypes are associated with different levels of haemolytic effect [12], i.e., individuals with South-East Asia variants or Middle East/West Asia variants have a stronger haemolytic response than individuals with the common African A- variants. Therefore, a long-term PQ dosing regimen for radical cure should be optimized according to patient’s G6PD activity status. For *P. falciparum* transmission-blocking regimen, the SLD-PQ is safe for G6PD-deficient individuals and it can be administered in all individuals without any G6PD testing [5].

This study evaluated the population pharmacokinetics of PQ, CPQ, and primaquine carbamoyl-glucuronide (PQCG) in G6PD-deficient African males. Also, the relationships between the pharmacokinetic parameters and the haemoglobin level in this population were evaluated.

## Methods

### Study design and ethical approval

This study was a part of an open-label, nonrandomized, dose-adjustment trial of the safety of SLD-PQ in G6PD-deficient and G6PD-normal males in Mali without microscopically detected malaria parasite infection. The main clinical trial was separated into two parts, i.e., Part I in adult males (aged 18–50) and Part II in children (aged 5–17). Full clinical details and results have been reported elsewhere [13]. Only 28 adult males from the main trial provided pharmacokinetic samples and were included in this study.

### Study procedure and blood sampling

After collection of day 0 samples, each participant received an oral dose of PQ according to his group assignment, after a fatty snack (biscuits) to minimized gastrointestinal symptoms. The study pharmacist prepared a dose by crushing a 15-mg tablet of PQ (Sanofi, Laval, Canada) in 15 mL of drinking water and administered the dose to the nearest 0.1 mL under direct observation. Venous samples (4 mL) were collected at pre-dose and 1, 4, 8, and 24 h post-dose. Capillary samples (0.5 mL) were collected at 2, 4, and 6 h post-dose. Both venous and capillary blood were collected using EDTA tubes, and centrifuged at 1100–1300×*g* for 10–20 min to obtain plasma samples. The samples were store immediately at − 80 °C until analysed.

### Bioanalytical methods

Only PQ and its metabolites, CPQ and PQCG, were detectable in plasma samples collected here. All compounds were analysed using the liquid chromatography–mass spectrometry (LC–MS) method of Avula et al*.* [14], modified to employ an ACQUITY UHPLC™, BEH Shield RP18 column (100 mm × 2.1 mm I.D., 1.7 mm) equipped with an LC-18 guard column (Vanguard 2.1 × 5 mm, Waters Corp, Milford, MA, USA). PQ, CPQ and PQCG were separated and eluted within 10-min retention time. The mobile phase, run at a flow rate of 0.25 ml/min, consisted of 0.05% formic acid in water (A) water and 0.05% formic acid in acetonitrile (B) and was applied in a linear gradient elution. The proportion of solvent A decreased from 90 to 63% during the first 5 min, then from 63 to 37% during minutes 5–8, then from 37 to 0% during minutes 8–10. A 3-min wash with 100% B and a 3.5 min equilibration period of 90% A followed each run. Samples were injected at 10 μL volume. The limits of quantification in plasma were 5 ng/mL for PQ and PQCG, and 1 ng/mL for CPQ. Quality control samples at 20 ng/mL were run at beginning and end of the batch analyses in duplicate. Intra-assay variability was less than 10%.

### Population pharmacokinetic model

PQ, CPQ, and PQCG concentrations were transformed into their natural logarithms and analysed using a nonlinear mixed-effects modelling approach in NONMEM version 7.4 (Icon Development Solution, Ellicott city, MD). Pirana version 3.0.0 [15], Perl-speaks-NONMEM version 5.3.0 (PsN) [16] and R version 4.2.0 were used for automation, model evaluation, and diagnostics during the model building process. The first-order conditional estimation method with interaction (FOCE-I) was applied for the estimation method. Each compound was modelled separately. Data below the LLOQ was either omitted (M1-method) or incorporated by imputing the first LLOQ data as half of LLOQ (M6-method) [17].

Pharmacokinetic parameters were implemented as a log-normal distribution as follows:

$$\theta_{i} = \theta_{{{\text{TV}}}} \times \exp \left( {\eta_{{i,{ }\theta }} } \right)$$

where ${\theta }_{i}$ is the individual $i$th pharmacokinetic parameter estimate, ${\theta }_{{\text{TV}}}$ is the typical value of the population mean estimate, and ${\eta }_{i, \theta }$ is the inter-individual variability of the parameter $\theta$ for the $i$th individual, assumed to be normal distribution with zero mean and variance ${\omega }^{2}$. Estimated inter-individual variability below 10% was fixed to zero.

A linear association between capillary and venous plasma concentrations of all compounds was assumed and modelled using an estimated conversion factor at the population level. Unexplained residual errors were modelled separately for capillary and venous plasma concentrations and implemented as an additive error model on the log-transformed concentrations, equivalent to an exponential error on arithmetic scale.

Individual body-weight (${{\text{BW}}}_{i}$) was introduced into the pharmacokinetic model as a fixed allometric function on all clearance ($n=0.75$) and volume ($n=1.00$) parameters, centralized to 62.5 kg of body weight according to median body weight in the population.

$$\theta_{i} = \theta_{{{\text{TV}}}} \times \exp \left( {\eta_{{i,{ }\theta }} } \right) \times \left( {\frac{{{\text{BW}}_{i} }}{62.5}} \right)^{n}$$

All other covariates of biological relevance (i.e. age, malaria status, G6PD genotypes, G6PD phenotypes, and CYP2D6 phenotype) were evaluated using a stepwise addition (*p* < 0.05) followed by a stepwise elimination (*p* < 0.001) approach.

### Model diagnostics and evaluations

The objective function value (OFV), calculated by NONMEM as proportional to − 2 × log-likelihood of the data, was used for evaluating the model fit. Any two hierarchical models were compared by a likelihood ratio test (LRT), based on the Chi-square distribution of OFV (i.e., p-value < 0.05 corresponding to ΔOFV > 3.48 at 1 degree of freedom difference). Potential model misspecification and systematic errors were determined by basic goodness-of-fit diagnostics. Eta and epsilon shrinkages were used to assess the ability of detecting model misspecification in the goodness-of-fit diagnostics [18]. Model robustness evaluation and generation of 95% confidence intervals were done using a sampling important resampling (SIR) procedure [19, 20]. Predictive performances of the final pharmacokinetic models were assessed by prediction-corrected visual predictive checks (n = 1000) [21]. The 5th, 50th and 95th percentiles were overlaid with the 95% confidence interval of each simulated percentile.

### Pharmacokinetic effects on haemoglobin and methaemoglobin levels

The potential relationship between individual pharmacokinetic parameters (i.e., estimated maximum concentration; C_MAX_, and 24-h exposure; AUC_24_) of all compounds and the changes in haemoglobin and methaemoglobin levels were characterised using a simple linear regression.

## Results

This was an open-label, Phase 1, dose-adjustment clinical trial in adults and children, and the main clinical findings have been reported previously [13]. Adult participants (n = 28) provided pharmacokinetic samples and were included in this study. Participants enrolled in this study were divided into 3 dosing groups, i.e., 0.40 mg/kg (n = 7), 0.45 mg/kg (n = 7), and 0.50 mg/kg (n = 14). Full demographic characteristics are presented in Table 1.

**Table 1:** Baseline subject’s characteristics

	Group 1 (n = 7) 0.40 mg/kg	Group 2 (n = 7) 0.45 mg/kg	Group 3 (n = 14) 0.50 mg/kg
Primaquine dose (mg)	24.0 (17.6–28.0)	26.1 (23.4–33.5)	32.1 (28.4–41.5)
Weight (kg)	60 (44.0–70.1)	57.9 (52.0–74.4)	64.1 (56.8–83.0)
Age (years)	20 (18–50)	32 (26–50)	39 (25.47)
Temperature (°C)	36.6 (36.5–37.4)	36.5 (36.1–37.2)	36.4 (36.1–37)
Haemoglobin (g/dL)	13.3 (12.0–15.5)	14.8 (14.4–17.5)	14.9 (13.6–16.5)
Positive malaria PCR (n)	5	5	4
G6PD genotype (SNPs 202A and 376G)			
Wild-type	3	2	7
A−	4	5	7
G6PD semi-quantitative^a^			
Normal	3	0	7
Deficient	4	6	7
CYP2D6 metabolism			
Poor metabolizer	1	0	0
Intermediate metabolizer	2	2	9
Extensive metabolizer	4	4	5
Extensive/ultra-rapid metabolizer	0	1	0

^a^G6PD testing results were obtained from a semi-quantitative test; haemoglobin levels of 0–6.5 U/g were considered as G6PD-deficient and haemoglobin levels > 6.5 U/g were considered as G6PD-normal

### Pharmacokinetic properties of PQ and its metabolites

Pharmacokinetic properties of PQ, CPQ, and PQCG were investigated separately. All 196 samples displayed concentrations above the LLOQ. Primaquine doses in molar units were used for metabolite dosing, under an assumption of 1:1 transformation factor.

**Fig. 1:**
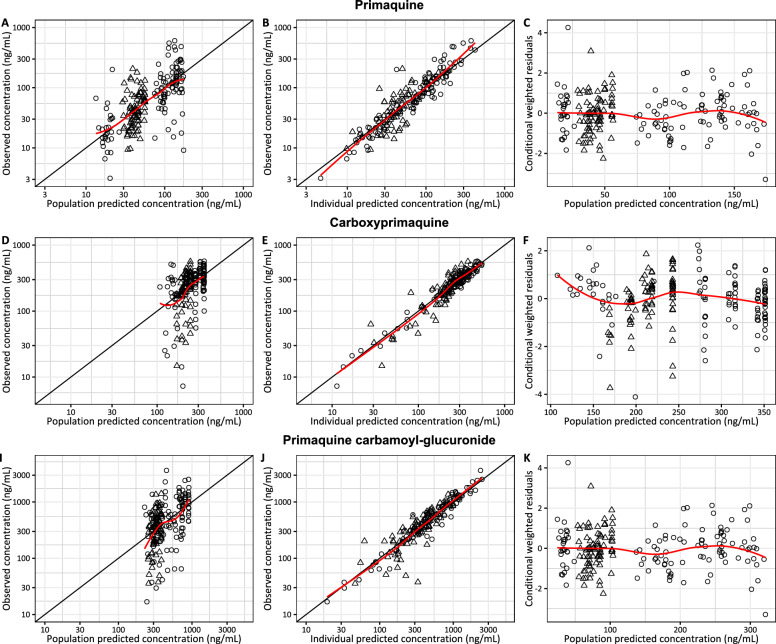
Goodness-of fit diagnostics of the final pharmacokinetic model of primaquine (**A**–**C**), carboxy-primaquine (**D**–**F**), and primaquine carbamoyl-glucuronide (**I**–**K**) stratified by biological matrix (i.e., circle = venous concentrations, and triangle = capillary concentrations). Red lines represent the locally weighted least-square regression based on the observations

**Fig. 2:**
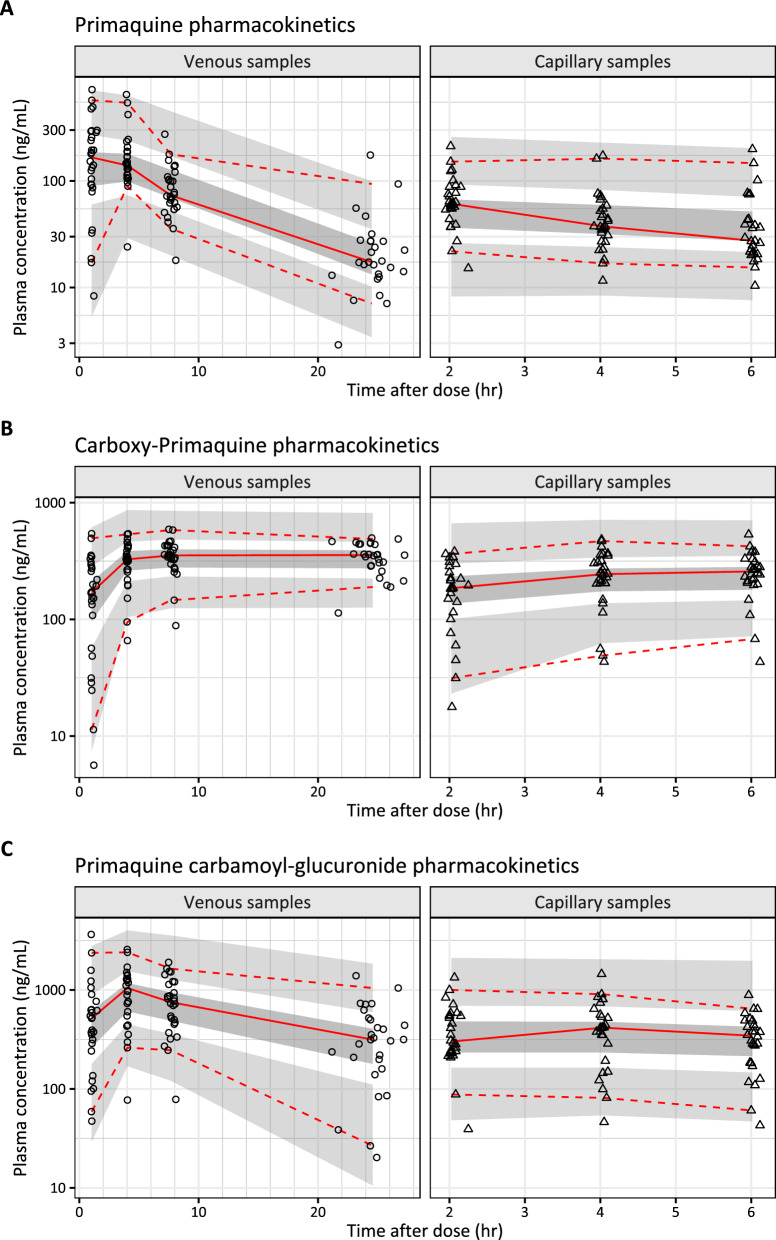
Visual predictive plots of the final pharmacokinetic model of primaquine (**A**), carboxy-primaquine (**B**), and primaquine carbamoyl-glucuronide (**C**). Solid and dashed lines represent the median, 5th, and 95th percentile of the observations. Shaded areas represent the simulated 95% confidence interval of each percentile

Pharmacokinetic properties of PQ, CPQ, and PQCG were explained by one-disposition compartment models with transit absorption models. Adding an additional peripheral distribution compartment did not improve the model fits. Absorption of PQ and PQCG were best described by 5 transit compartment models, whereas the absorption of CPQ was best described by a 2 transit compartment model. Body-weight was added into the pharmacokinetic model as an allometric function. None of the other covariates were statistically significant. The G6PD genotype (A-variant and wild-type) and G6PD phenotype (determined by a semiquantitative test) were not statistically significant on any pharmacokinetic parameters of PQ or its metabolites. The final models of all compounds showed a satisfactory goodness-of-fit (Fig. 1) and predictive performance (Fig. 2). The venous-capillary conversion factors for all compounds were estimated using the final population pharmacokinetic models. Also, the estimated conversion factors agreed with a simple linear-regression model using measured concentrations of samples taken at the same time point (Figure S1). The final pharmacokinetic parameter estimates with its parameters’ uncertainty and the secondary parameter estimates are reported in Tables 2 and 3, respectively.

**Table 2:** Final pharmacokinetic parameter estimates

Pharmacokinetic parameter	Population estimate^a^ (%RSE^b^)	95% CI^b^	IIV^a^ (%CV^b^)	95% CI^b^
Primaquine				
F	1 fixed	–	52.9% (15.4%)	39.7%–74.6%
MTT (h)	0.563 (18.7%)	0.329–0.754	63.3% (23.5%)	36.8%–100%
CL/F (L/h)	15.4 (9.57%)	13.1–18.7	12% (12.4%)	7.99%–14.3%
V_C_/F (L)	163 (10.3%)	134–201	–	–
CF (%)	32.9 (6.89%)	28.7–37.5	–	–
σ_VP_	0.173 (9.37%)	0.125–0.245	–	–
σ_CP_	0.226 (9.33%)	0.157–0.320	–	–
Carboxy-primaquine				
MTT (h)	1.24 (11.7%)	0.962–0.746	65.3% (13.2%)	50.6%–89.2%
CL/F (L/h)	0.129 (28.5%)	0.0765–0.216	–	–
V_C_/F (L)	93.3 (7.00%)	81.8–108	37.4% (15.7%)	29.5%–52.9%
CF (%)	69.1 (3.83%)	0.643–0.746	–	–
σ_VP_	0.0328 (8.96%)	0.0238–0.0474	–	–
σ_CP_	0.101 (8.84%)	0.0763–0.146	–	–
Primaquine carbamoyl-glucuronide				
F	1 fixed	–	63.5% (12.1%)	48.7%–81.9%
MTT (h)	1.13 (8.84%)	0.929–1.33	34.4% (25.1%)	24.2%–58.5%
CL/F (L/h)	2.83 (15.8%)	2.06–3.81	57.8% (12.1%)	42.8%–75.1%
V_C_/F (L)	55.4 (12.4%)	44.1–72.2	–	–
CF (%)	40.1 (6.97%)	0.353–0.465	–	–
σ_VP_	0.108 (12.3%)	0.0736–0.175	–	–
σ_CP_	0.242 (8.00%)	0.178–0.323	–	–

CF is the proportional conversion factor between capillary and venous drug measurements, CL/F is the apparent elimination clearance, F is the relative bioavailability, MTT is the absorption mean transit time, V_C_/F is the apparent volume of distribution of the central compartment, σ_CP_ is the variance of an exponential residual error of the capillary samples, and σ_VP_ is the variance of an exponential residual error of the venous samples

^a^Computed population mean parameter estimates from NONMEM were calculated for a typical individual at a body weight of 62.5 kg. The coefficient of variation (%CV) of the inter-individual variability (IIV) was calculated as $100\times \sqrt{{\text{exp}}\left({\omega }^{2}\right)-1}$

^b^Computed from the sampling important resampling (SIR) procedure [19, 20] of the final pharmacokinetic models

**Table 3:** Secondary pharmacokinetic parameter estimates

Parameter	Primaquine	Carboxy-primaquine	Primaquine carbamoyl-glucuronide
C_MAX_ (ng/mL)	259 (53.5–764)	338 (74.3–546)	1550 (246–7320)
T_MAX_ (h)	0.872 (0.237–1.77)	4.57 (2.19–18.1)	1.61 (0.572–1.78)
AUC_24_ (h × ng/mL)	130 (59.2–491)	8030 (1490–12,800)	1240 (242–3100)
Half-life (h)	7.42 (7.04–7.93)	469 (325–1870)	17.5 (11.1–28.0)

Numbers are presented as median (range)

AUC_24_ is the area under the concentration–time curve up to 24 h, C_MAX_ is the maximum concentration, and T_MAX_ is the time to maximum concentration

### Effect of pharmacokinetic parameters on haemoglobin

**Fig. 3:**
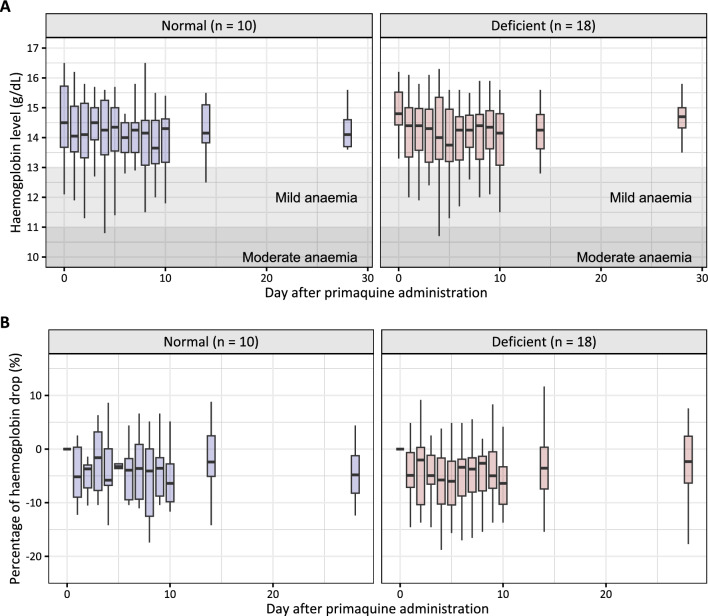
Observed haemoglobin concentration (**A**) and percentage of haemoglobin drop from the baseline (**B**) over 28 days of follow-up, stratified by G6PD status. Shaded areas represent a mild and moderate anaemia

**Fig. 4:**
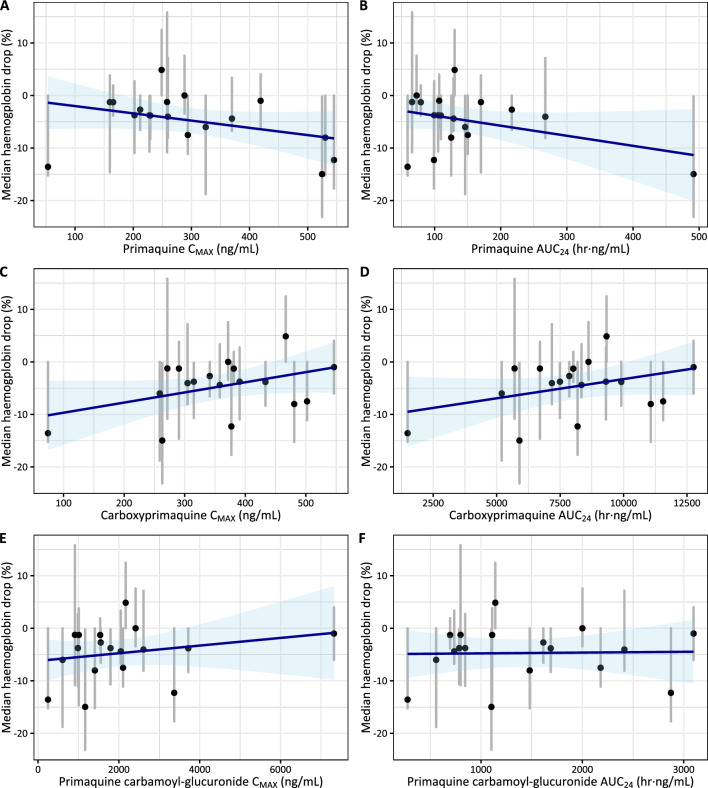
Correlation between pharmacokinetic parameters (C_MAX_ and AUC_24_) and the median haemoglobin drop for primaquine (**A**, **B**), carboxy-primaquine (**C**, **D**), and primaquine cabarmoyl-glucuronide (**E**, **F**) in G6PD-deficient individuals (n = 18). Blue lines and shaded areas represent the linear regression with associated prediction intervals. Black circles and grey lines represent individual’s mean and range of observed haemoglobin drop. None of these correlations are significantly different from the zero-slope

**Fig. 5:**
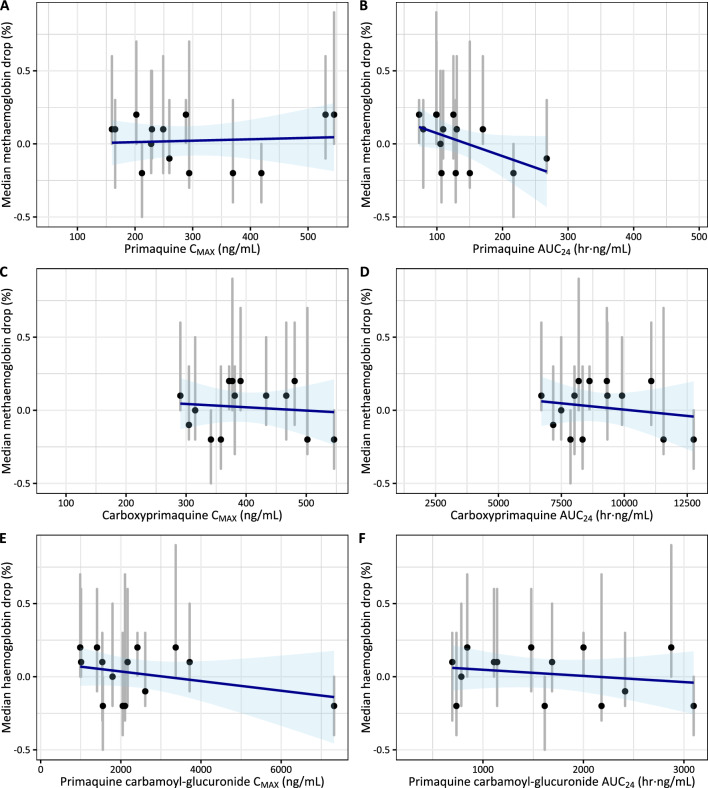
Correlation between pharmacokinetic parameters (C_MAX_ and AUC_24_) and the median haemoglobin drop for primaquine (**A**, **B**), carboxy-primaquine (**C**, **D**), and primaquine cabarmoyl-glucuronide (**E**, **F**) in G6PD-deficient individuals (n = 18). Blue lines and shaded areas represent the linear regression with associated prediction intervals. Black circles and grey lines represent individual’s mean and range of observed methaemoglobin drop. None of these correlations are significantly different from the zero-slope

Haemoglobin and methaemoglobin levels were observed for up to 28 days after PQ administration (Fig. 3, Figure S2). There were no significant haemoglobin declines in patients with G6PD deficient and G6PD-normal status. Suggestive trends were observed between the pharmacokinetic parameters of PQ and the small decreases observed in haemoglobin levels (Fig. 4, Table S1) or increases in methaemoglobin levels (Fig. 5, Table S2). However, these relationships were not statistically significant from the zero-correlation and not statistically significant different between the G6PD-deficient and G6PD-normal individuals.

## Discussion

Pharmacokinetic properties of PQ and CPQ estimated in this study were similar to previous literature reports [2, 7, 22]. Pharmacokinetic properties of the carbamoyl-glucuronide metabolite are reported here for the first time.

Pharmacokinetic models of PQ and its metabolites were fitted separately due to the unknown fraction of total primaquine elimination resulting in each specific metabolic pathway. Metabolism of PQ is quite complex which several enzymes and intermediate metabolites involved [8]. CPQ is a stable abundant metabolite mediated by monoamine oxidase A (MAO-A) and it’s commonly measured together with the parent drug. On the other hand, PQ is also metabolized by the cytochrome P450 2D6 (CYP2D6) isoform. Quinone-imine and orthoquinone metabolites can generate a reactive oxygen species through redox cycling, and might be therefore responsible for antiparasitic activity and haemotoxicity. Drug-metabolite models of PQ and CPQ have been reported previously, both as empirical and mechanistic population pharmacokinetic models [7, 23, 24]. One-compartment disposition kinetic of PQ and CPQ reported here were congruent with previous studies. PQ was estimated to have a short terminal elimination half-life of approximately 7.42 h, while its carboxy metabolite had a longer terminal elimination half-life of approximately 19.5 days. This estimated terminal half-life of CPQ here was substantially longer than that previously reported in healthy volunteers (19.5 days vs 15.6 h) [7]. This might be explained by a short period of sampling times in previous studies (i.e. within 24 h); resulting in capturing mostly the absorption/distribution phase and not the terminal elimination phase, and thereby underestimating the true half-life of CPQ. Disposition kinetics of PQCG was explained best by using a one-compartment disposition model and resulted in an estimated terminal elimination half-life of 17.5 h. Implementation of CYP2D6 or G6PD status did not improve the pharmacokinetic model significantly.

As expected, it was a close correlation (1:1) between capillary plasma and venous plasma concentrations of PQ, CPQ and PQCG [25]. In this study, the conversion factor between capillary and venous plasma concentrations of PQ, CPQ, and PQCG were estimated at 32.9%, 69.1%, and 40.1%, respectively. This is not entirely consistent with literature, which report a capillary to venous plasma ratio of approximately 1:1 for PQ and CPQ [25]. This needs to be investigate further to elucidate potential differences in capillary vs venous plasma measurements in different populations and settings.

The WHO suggests to add PQ to the standard ACT for the treatment of uncomplicated falciparum malaria in order to block further malaria transmission [1]. This SLD-PQ (0.25 mg/kg) has been shown to be sufficient for reducing gametocyte carriage and preventing malaria transmission [2, 26]. The same dose is also shown to be safe in G6PD-deficient individuals and does not require prior G6PD activity testing [5]. This study showed that the SLD-PQ is associated with a small increment of oxidative stress and low risk of haemolytic events in the G6PD-deficient individuals. However, a long-term PQ administration (15 mg daily for 14 days) in the G6PD-deficient individuals is associated with a high risk of acute haemolytic anaemia, and once weekly dosing for 8 weeks is recommended in these individuals. A mathematical modelling study proposed that a daily escalating PQ dose in G6PD-deficient individuals might be safer than the current weekly dosing regimen [27]. The predicted median reduction of haemoglobin after 0.25 and 0.40 mg/kg SLD-PQ in G6PD-deficient individual was 0.35 g/dL (90% CI 0.12–0.65 g/dL) and 0.56 g/dL (90% CI 0.21–1.00 g/dL), respectively [28].

This study has several limitations. Pharmacokinetic data were collected only for 24 h after drug administration, and the sampling schedule did not capture fully the elimination phase of CPQ. A pharmacokinetic study with longer duration of pharmacokinetic sampling and more data in the elimination phase of CPQ could improve the accuracy of the estimated parameters. PQ enantiomers have been shown to exhibit different pharmacokinetic profiles [6, 7, 9], but the bioanalytical assays used here could not distinguish between different enantiomers. Thus, the racemic mixture of PQ was modelled and no conclusions can be drawn on the contribution of individual enantiomers. The metabolism pathways of PQ are quite complex with several inactive compounds involved [8], and only few of these compounds can be quantified in clinical samples. More specifically 5,6-orthoquinoline, the metabolite believed to be responsible for adverse reactions and linked to clinical outcome [11], cannot be measured in human plasma. Therefore, the association between PQ and the active orthoquinone compound has not been well characterized.

## Conclusions

In summary, the population pharmacokinetic properties of PQ, CPQ, and PQCG have been characterized and reported here. No statistically significant relationships were seen between the pharmacokinetic parameters and the change in haemoglobin levels in G6PD-deficient patients after a single low dose of primaquine. A single low dose (0.50 mg/kg) of PQ was haematologically safe in this population of G6PD-deficient African males without malaria.

### Supplementary Information

Supplementary Material 1.

## Data Availability

Deidentified participant data will be available after publication to applicants who provide a sound proposal to the Mahidol Oxford Tropical Medicine Research Unit Data Access Committee. They can contact the corresponding author in the first instance.

## Abbreviations

%CV: Percentage of coefficient of variation
%RSE: Percentage of Relative standard error
ACT: Artemisinin combination treatment
CPQ: Carboxyprimaquine
CYP: Cytochrome P450
G6PD: Glucose-6-phosphate dehydrogenase
IIV: Inter-individual variability
LLOQ: Lower limit of quantification
LRT: Likelihood ratio test
MAO-A: Monoamine oxidase
OFV: Objective function value
PQ: Primaquine
PQCQ: Primaquine carbamoyl-glucuronide
SIR: Sampling important resampling
SLD: A single low dose
UDPGA: Uridine-diphosphate-glucuronic-acid
