# Supplementary material for: Population pharmacokinetics of primaquine and its metabolites in African males
Source: Malar J. 2024 May 21;23:159. doi: 10.1186/s12936-024-04979-y (PMC11106956; doi:10.1186/s12936-024-04979-y)
Supplement: Supplementary file 1 — Supplementary Material 1. [file 12936_2024_4979_MOESM1_ESM.docx]

# Supplementary Material

**Population pharmacokinetics of primaquine and its metabolites in African males**

*P. Chotsiri, A. Mahamar, H. Diawara, P.S. Fasinu, K. Diarra, K. Sanogo, T. Bousema, L.A. Walker, J.M. Brown, A. Dicko, R. Gosling, I. Chen, J. Tarning.*


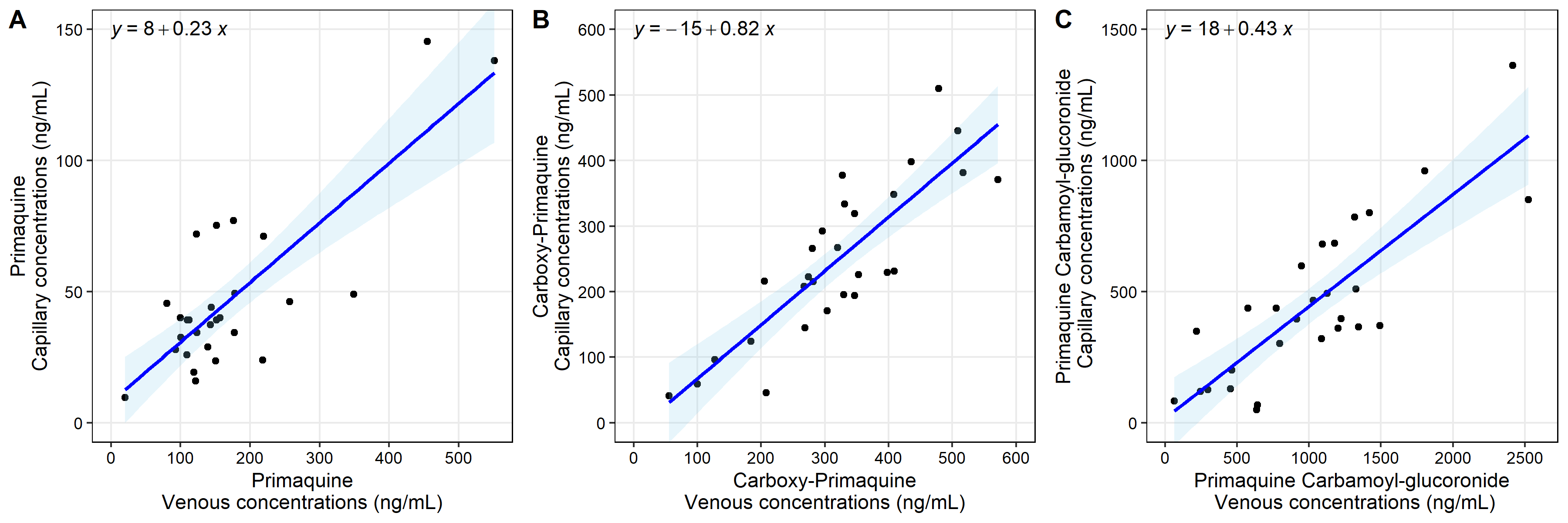


**Figure S1.** Correlation between capillary plasma and venous plasma concentrations using the time-match observations of primaquine (A), carboxy-primaquine (B), and primaquine carbamoyl-glucuronide (C). Blue lines and shaded areas represent a linear correlation and its 95% confidence intervals of the venous and capillary concentrations, respectively.


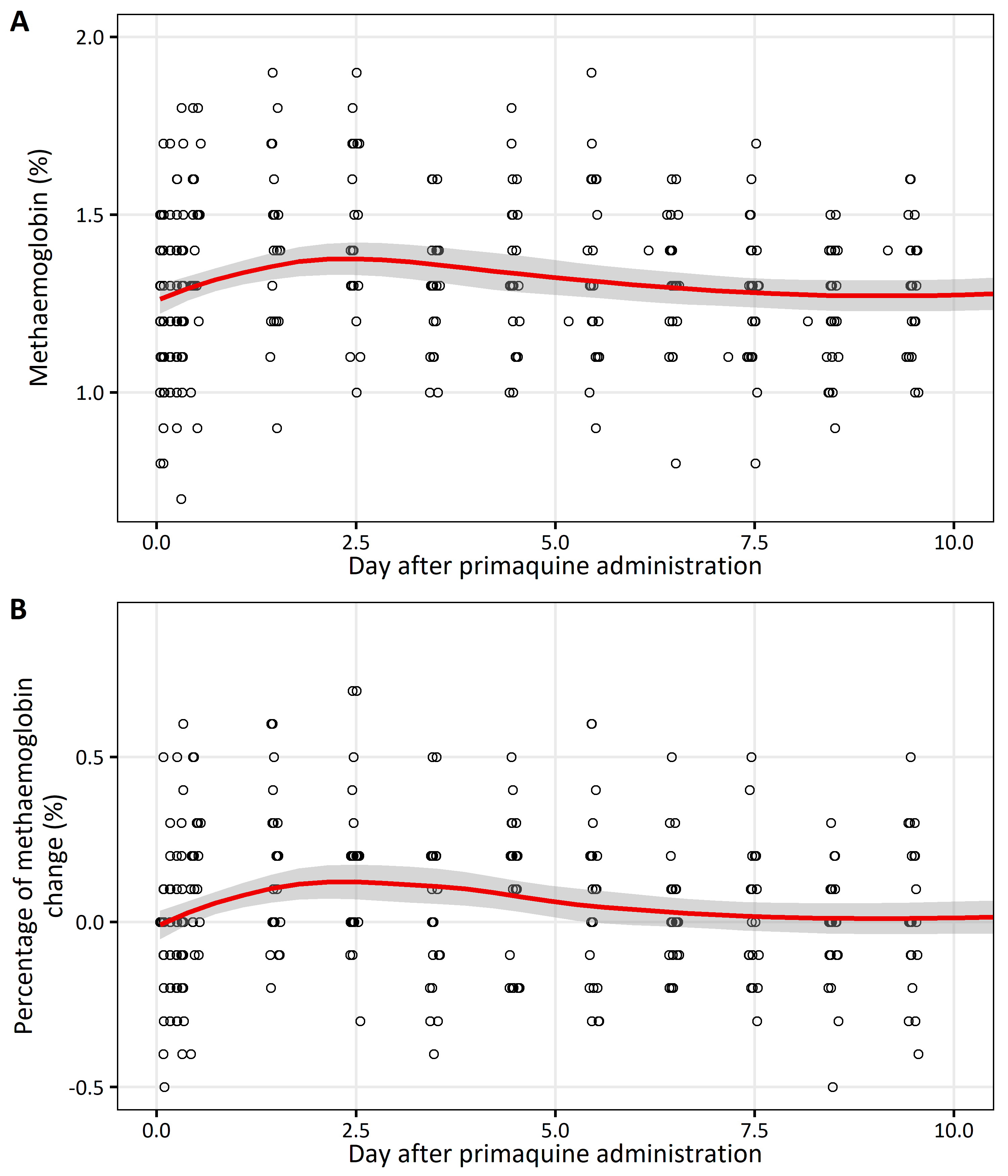


**Figure S2.** Observed methaemoglobin concentration (A) and percentage of methaemoglobin drop from baseline (B) over 10 days of follow-up. Red lines and shaded areas represent the locally weighted least-square regression based on the observations and its 95% confidence intervals, respectively.


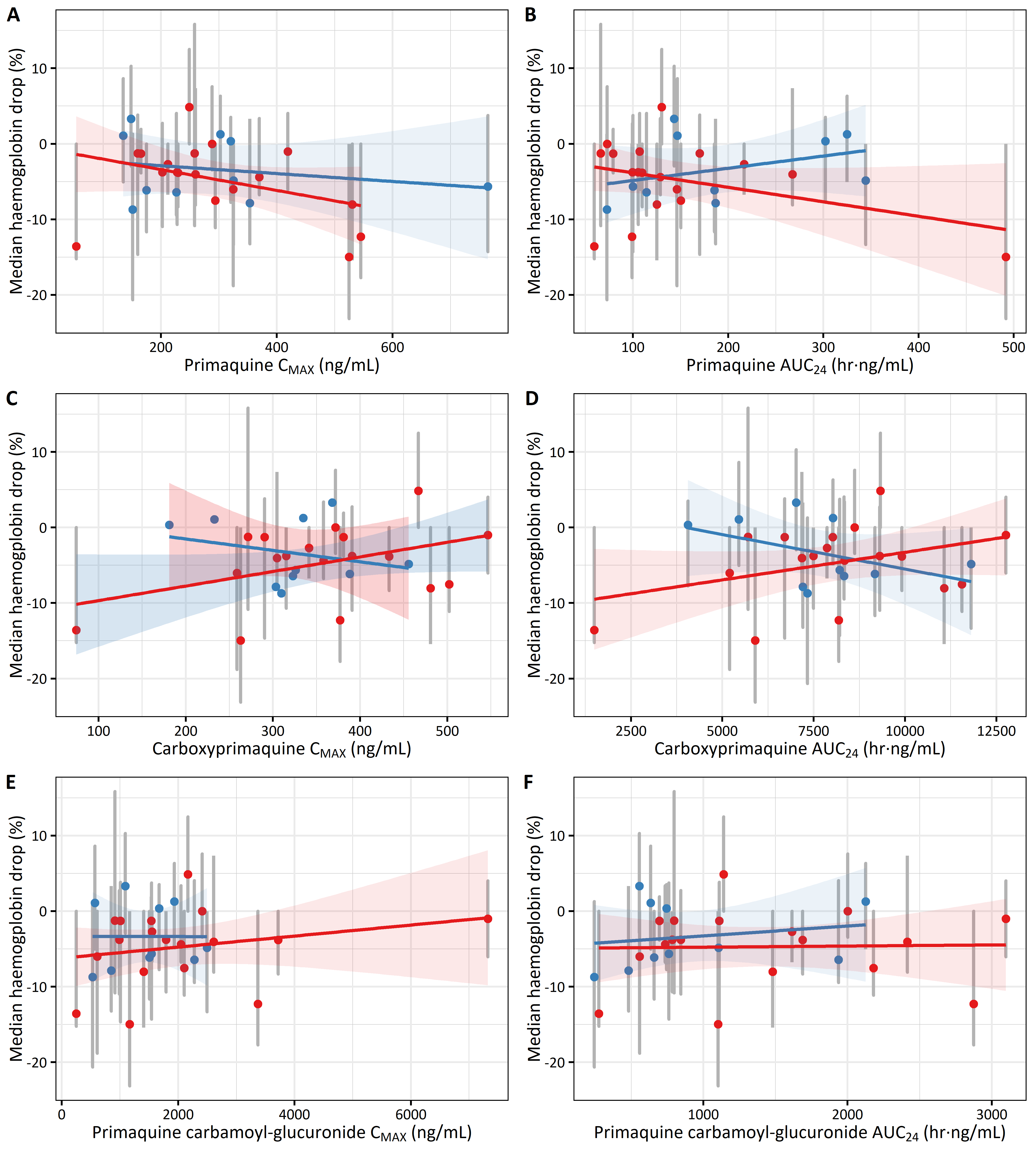


**Figure S3.** Correlation between pharmacokinetic parameters (C_MAX_ and AUC_24_) and the maximum haemoglobin drop for primaquine (A, B), carboxy-primaquine (C-D), and primaquine cabarmoyl-glucuronide (E-F) in G6PD-deficient individuals (red) and G6PD-normal individuals (blue). None of these correlations showed a significant difference from the zero-slope. Lines and shaded areas represent the predicted linear correlation and its 95% confidence intervals, respectively.


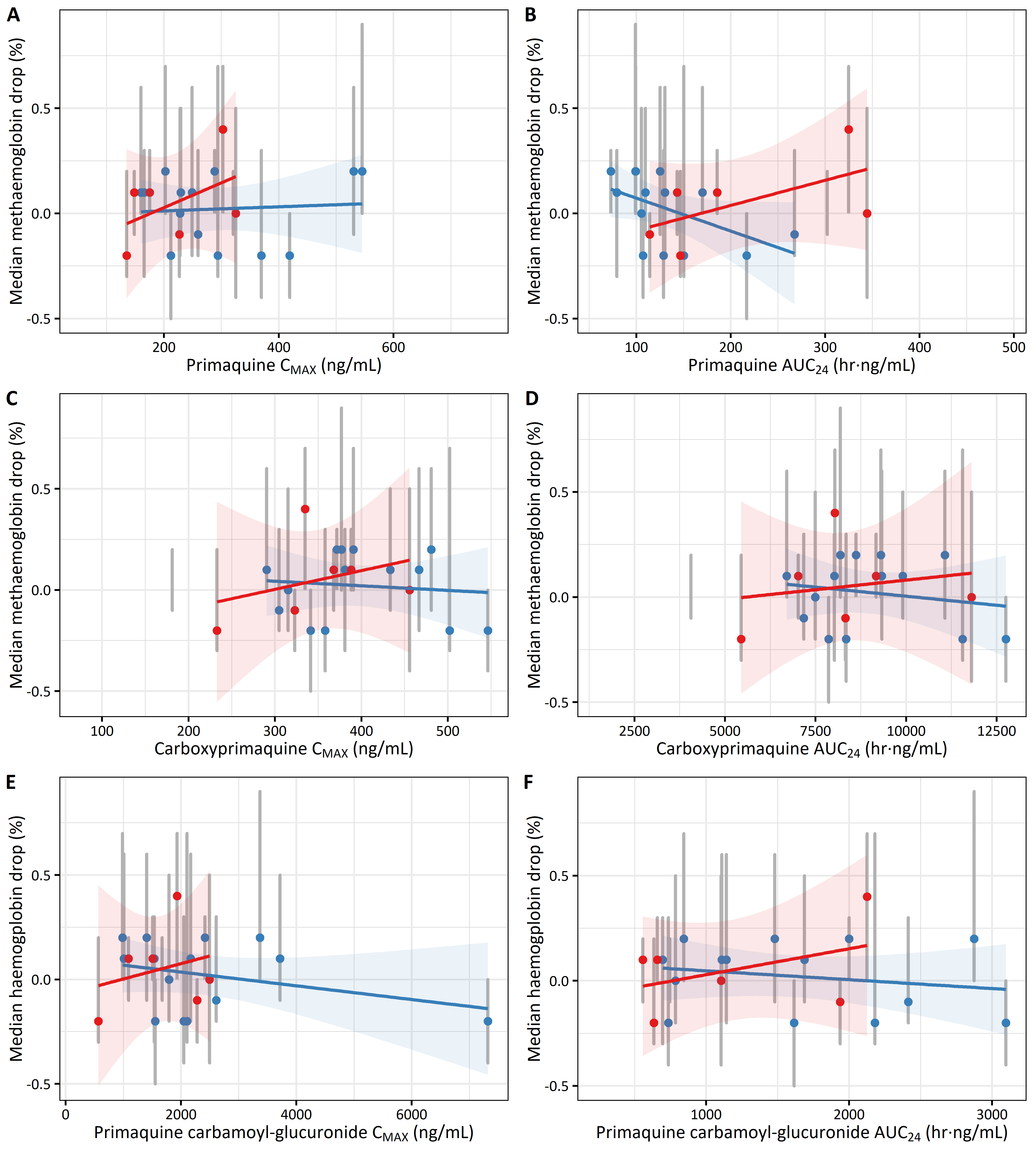


**Figure S4.** Correlation between pharmacokinetic parameters (C_MAX_ and AUC_24_) and the maximum methaemoglobin change for primaquine (A, B), carboxy-primaquine (C-D), and primaquine cabarmoyl-glucuronide (E-F) in G6PD-deficient individuals (red) and G6PD-normal individuals (blue). None of these correlations showed a significant difference from the zero-slope. Lines and shaded areas represent the predicted linear correlation and its 95% confidence intervals, respectively.

**Table S1.** Correlation between pharmacokinetic parameters and the change in haemoglobin.

|  | **G6PD-deficient individuals  (n = 18)** | **G6PD-normal individuals  (n = 10)** |
| --- | --- | --- |
| *Primaquine* |  |  |
| **C_MAX_** (% HB change per 1 ng/mL) | -10.1 (95% CI: -23.3 to 3.21)  *p* = 0.127 | -9.41 (95% CI: -43.2 to 24.4)  *p* = 0.539 |
| **AUC_24_** (% HB change per 1 ng⋅hr/mL) | -7.69 (95% CI: -17.5 to 2.11)  *p* = 0.116 | 8.10 (95% CI: -8.89 to 25.1)  *p* = 0.304 |
| *Carboxy-primaquine* |  |  |
| **C_MAX_** (% HB change per 1 ng/mL) | 9.27 (95% CI: -1.25 to 19.8)  *p* = 0.0803 | -4.63 (95% CI: -18.4 to 9.13)  *p* = 0.460 |
| **AUC_24_** (% HB change per 1 ng⋅hr/mL) | 195 (95% CI: -58.7 to 448)  *p* = 0.123 | -206 (95% CI: -555 to 143)  *p* = 0.210 |
| *Primaquine carbamoyl-glucuronide* |  |  |
| **C_MAX_** (% HB change per 1 ng/mL) | 73.3 (95% CI: -90.1 to 237)  *p* = 0.356 | -0.377 (95% CI: -127 to 127)  *p* = 0.995 |
| **AUC_24_** (% HB change per 1 ng⋅hr/mL) | 3.91 (95% CI: -82.1 to 89.9)  *p* = 0.924 | -26.3 (95% CI: -88.0 to 141)  *p* = 0.610 |

*G6PD is glucose-6-phosphate dehydrogenase, C_MAX_ is the maximum drug concentration, AUC_24_ is the daily area under the drug concentration-time profile, and HB is the haemoglobin.*

**Table S2.** Correlation between pharmacokinetic parameters and the change in methaemoglobin.

|  | **G6PD-deficient individuals  (n = 18)** | **G6PD-normal individuals  (n = 10)** |
| --- | --- | --- |
| *Primaquine* | | |
| **C_MAX_** (% mHB change per 1 ng/mL) | 54.1 (95% CI: -413 to 531)  *p-value* = 0.252 | -177 (95% CI: -302 to 656)  *p-value* = 0.363 |
| **AUC_24_** (% mHB change per 1 ng⋅hr/mL) | -162 (95% CI: -337 to 12.6)  *p-value* = 0.0661 | 275 (95% CI: -271 to 821)  *p-value* = 0.234 |
| *Carboxy-primaquine* | | |
| **C_MAX_** (% mHB change per 1 ng/mL) | -49.4 (95% CI: -341 to 242)  *p-value* = 0.719 | 119 (95% CI: -351 to 589)  *p-value* = 0.521 |
| **AUC_24_** (% mHB change per 1 ng⋅hr/mL) | -1914 (95% CI: -8430 to 4602)  *p-value* = 0.534 | 1948 (95% CI: -12127 to 16023)  *p-value* = 0.720 |
| *Primaquine carbamoyl-glucuronide* | | |
| **C_MAX_** (% mHB change per 1 ng/mL) | -3071 (95% CI: -8834 to 2691)  *p-value* = 0.268 | 922 (95% CI: -3823 to 5667)  *p-value* = 0.618 |
| **AUC_24_** (% mHB change per 1 ng⋅hr/mL) | -961 (95% CI: -3904 to 1982)  *p-value* = 0.490 | 1396 (95% CI: -2851 to 5643)  *p-value* = 0.413 |

*G6PD is glucose-6-phosphate dehydrogenase, C_MAX_ is the maximum drug concentration, AUC_24_ is the daily area under the drug concentration-time profile, and mHB is the methaemoglobin.*
